# Anaesthetic Management of Carinal Resection and Reconstruction–A Case Report

**Published:** 2009-06

**Authors:** TVSP Murthy

**Affiliations:** Prof and Senior Adviser and HOD

**Keywords:** Carinal resection, General anesthesia, Pneumonectomy, Bronchopleural fistula

## Abstract

**Summary:**

Anaesthetic management of surgery for tumors involving the carina is very challenging for an anaesthesiologist and has been associated with guarded prognosis. We describe the management of carinal resection and reconstruction due to a tracheal tumor involving the carina. The various anaesthetic issues involved and experienced in this clinical setting are described.

## Introduction

Despite recent technical refinements in tracheal surgery and bronchial sleeve lobectomy, tumors with involvement of the carina still remain a challenge for both thoracic surgeons and anaesthesiologists^1^. The anaesthetic management of a patient who underwent carinal and sleeve right main bronchus resection with a right sided pneumonectomy for an endobronchial malignancy with non small cell lung tumor performed through right thoracotomy is presented^2^.

## Case Report

A 38-year-old, female patient, presented with a history of fever, chills, cough and one attack of hemoptysis. CT-scan of the chest revealed an endobronchial lesion obstructing the right main stem bronchus. A bronchoscopy was performed which showed a tumor involving the right main stem bronchus, encroaching into the midline above the carina. A biopsy was positive for the malignancy. Routine laboratory investigations were normal. Pulmonary function tests showed an FEV1 of 1.47 liter (54% predicted), FVC of 1.7 liter (52% predicted) and FEV1/FVC 86%. There was moderate to severe reduction of lung volumes with normal diffusion capacity. Investigations revealed normal ECG, bone scan and ultra sound of the abdomen. Her chest x-ray and CT scan of the chest confirmed the diagnosis.

The patient was scheduled for carinal resection, right sided pneumonectomy and anastomosis of lower end of trachea with left main bronchus through a right thoracotomy.

Preoperative assessment revealed well looked lady with shortness of breath, but was not distressed. There was no lymphadenopathy or clubbing. Vital signs were normal. Chest examination revealed slightly deviated trachea to the left side, and normal breath sounds on both sides. Cardiovascular system was normal. Abdomen was soft, lax with no organomegaly. Arterial blood gases (ABG) on room air showed, PaO_2_ 166 mmHg, PaCO_2_ 42 mmHg and O_2_ saturation 94%.

After an informed consent, she was counseled on the risks involved in the procedure and was later accepted for the surgery under general anaesthesia in ASA Grade III. In the operating room cardio respiratory monitoring was instituted. Intravenous and arterial lines were established. A thoracic epidural catheter under local analgesia was inserted at D 7-8, to take care of post operative pain relief. After preoxygenation anesthesia was induced with intravenous fentanyl 2 mcg.kg^−1^ and propofol 200 mg, and inhalation via mask with oxygen, nitrous oxide and sevoflurane. Suxamethonium 100 mg was given to facilitate endotracheal intubation which was achieved with a left sided 35 size double lumen endotracheal tube. The intubation was done carefully under vision using a 4mm fibreoptic bronchoscope so as to avoid any bleed from the tumour site and to confirm its correct placement. The left-side internal jugular vein was later cannulated for central venous pressure monitoring, the right side was deliberately avoided as it comes in the field of the surgery and the surgeon had requested for the same. The left side cannulation is comparatively more difficult as compared to the right, but attempted carefully is also a safe alternative. Anaesthesia was maintained with 50% N2O in O2 and 2% sevoflurane. The patient was positioned on left lateral position and a right posterolateral thoracotomy was performed.

Once thoracotomy was done the right lung ventilation was cut off allowing the lung to collapse to facilitate surgical procedure of pneumonectomy. The ventilatory settings were adjusted so as to avoid any barotraumas or unacceptable high airway pressure. The saturations were maintained using low tidal volume and high respiratory rate. After mobilization of the lower trachea, carina, and both main bronchi, resection of the carina and 2 cm of the proximal left main bronchus was performed.

Before the resection of the proximal left main bronchus, the left main bronchus was intubated across the operative field with a sterile flexometallic endotracheal tube (6.0 mm ID), with sterile connecting tubing and ventilation maintained, with around 300 ml of tidal volume and a check on airway pressure not to increase beyond 25 mmHg and achieve a end tidal CO_2_ of about 35–40 mmHg by adjusting the respiratory rate.

During the anastomosis a close cooperation existed between the anaesthesiologist and surgeon with intermittent ventilation of the lung alternating with temporary removal of the tube for precise placement of anastamotic sutures. Ventilation during this time was maintained with apnoeic oxygenation. A close watch on end tidal carbon dioxide and oxygen saturation levels helped keep the respiratory and haemodynamic parameters in acceptable range, by adjusting the ventilatory settings as described above.

After two thirds of the anastomosis between the trachea and the left main bronchus has been completed, the double lumen tube is advanced into the left main bronchus again under vision. The flexometallic tube in the left main bronchus is later removed and wound repaired. Significant reduction in anastmotic tension is achieved by the mobilization of the hilum with an inferior hilar release on either side.

Once the resection and anastomosis was completed the double lumen endotracheal tube was removed and a conventional flexometallic single lumen endotracheal tube reinserted under vision over the fiber optic bronchoscope. At the end of surgery the patient was reversed with atropine/neostigmine (0.5/2.5 mg) and the endotracheal tube was left in situ for airway protection. To avoid any untoward tension on the anastmotic site the chin of the patient was sutured to the anterior chest wall ensuring a slight flexion and avoidance of extension at the neck.

The patient was transferred to the surgical intensive care unit for further observation. Post operatively she was maintained on both epidural analgesia with fentanyl and bupivacaine (500 μgm of fentanyl and 0.125% concentration of Bupivacaine – in a 250ml elastomeric balloon pump, delivered at the rate of 5ml/hr) and also systemic sedation with midazolam so as to tolerate the endotracheal tube with any exertion. She made uneventful recovery and eventually was extubated on the second day following which she was later discharged to the surgical ward from the intensive care.

## Discussion

Despite sporadic reports in the literature over the past many years, carinal resection remains a relatively daunting and infrequently used procedure for most thoracic surgeons.

It is well known that resection of the tracheobronchial tree is indicated in patients who have tracheal obstruction due to primary tracheal tumor, tracheal stenosis, congenital anomalies, and vascular lesions^3^.

In the present case report the tumor originated from the right main bronchus and involved the carina. Ventilation and maintaining oxygenation during tracheobronchial resection surgery is the challenging moment for the anaesthesiologist. A variety of methods for providing adequate oxygenation and carbon dioxide elimination have been used during tracheal resection^4^. These include, standard orotracheal intubation, insertion of a tube into the opened trachea distal to the area of re-section, high frequency jet ventilation (HFJV) through the stenotic area, low frequency jet ventilation for stent insertion, high frequency positive pressure ventilation (HFPPV), and cardiopulmonary bypass specially when left thoracotomy approach is used^5^,^6^. High-frequency jet ventilation represents the optimal modality of ventilation for surgery of the distal portion of the trachea. In any case, a sterile standard endotracheal tube must be promptly available to ventilate the patient through the operative field if high-frequency jet ventilation becomes insufficient, especially in patients with poor pulmonary function.

The anticipated technical limitations to the performance of tracheobronchial surgery can now be overcome by careful preoperative assessment of the site and degree of obstruction, close intraoperative communication between the surgeon and anaesthesiologist, improved anaesthetic management techniques, and intensive post operative care.

It is well known that the ability to provide adequate ventilation throughout the perioperative period is a major predictor for better outcome during tracheal resection surgery^7^. Though not resorted to tracheostomy in many of these surgeries postoperatively it has well-known benefits including the facilitation of secretion removal and decreasing the work of breathing by reduction of dead space ventilation and hence requires serious consideration^8^.

The predominant predictors of postoperative morbidity and mortality include postoperative mechanical ventilation, the extent of airway resection, and the development of anastamotic complications^9^. Mechanical ventilation predisposes to barotrauma and the development of infectious pulmonary complications and is detrimental to airway healing. Postoperative ventilation has been previously shown as a risk factor for the development of bronchopleural fistula especially after pneumonectomy.^10^,^11^,^12^

Understanding the safe limits of resection, the technical advances of airway reconstruction, and methods to reduce anastamotic tension should minimize the serious problem of anastamotic morbidity which was associated with a 43% mortality rate.^13^.

Adult respiratory distress syndrome could develop as a consequence of pneumonectomy and surgery in few cases which will significantly impact the outcome of patients after particularly carinal pneumonectomy. Successful management of this lethal problem after pneumonectomy has been reported with the use of nitric oxide and may be with extra pulmonary ventilation if required^14^.

Every attempt should be made, to extubate these patients at the end of the procedure which should be achievable in most patients, as ventilation could lead to a possible anastamotic leak, barotraumas and a high incidence of developing a bronchopleural fistula. Earlier reports of carinal surgery stressed that the extent of airway resection should be limited to less than 4 cm to minimize the risk of an anastamotic complication when the trachea is to be reconnected end to end with the left main bronchus^1^,^15^. This is due primarily to the relative immobility of the left bronchus, which is tethered in it's cephalad migration by the aortic arch. The lack of mobility may result in excessive tension on the anastomosis. These limitations are not applicable to anastomosis involving the trachea end to end with the right main bronchus, which can be mobilized extensively with a full right hilar release^15^. The anaesthesiologist should bear these issues in mind so that in case of any ventilatory support required he should be guarded in providing ventilation so as to avoid complications listed above.

Tumors with involvement of the carina remain a rare condition, but surgical resection can be proposed in selected patients with acceptable morbidity and mortality rates. Surgical resection of lung or tracheal tumors with carinal involvement being unusual condition remains a challenge for the anaesthesiologist. The case is presented to share the implications involved in anaesthesia during the management of case which are not very common in day to day anesthetic practice.
